# Antibiotic prescribing trends and outcomes in outpatient low-risk diverticulitis: a retrospective analysis in Veterans Health Administration between 2016 and 2023

**DOI:** 10.1017/ash.2026.10884

**Published:** 2026-10-01

**Authors:** Shinya Hasegawa, Elizabeth A. Chrischilles, Tomohiro Tanaka, Hiroyuki Suzuki

**Affiliations:** 1 Division of Infectious Diseases, Department of Internal Medicine, University of Iowa, Iowa City, IA, USA; 2 Center for Access and Delivery Research and Evaluation, Iowa City Veterans Affairs Health Care System, Iowa City, IA, USA; 3 Department of Epidemiology, University of Iowa, Iowa City, IA, USA; 4 Division of Gastroenterology and Hepatology, Department of Internal Medicine, University of Iowa, Iowa City, IA, USA

## Abstract

**Background:**

Outpatient antibiotic prescribing remains common despite evidence that selective non-antibiotic management is safe for low-risk uncomplicated diverticulitis. Although recent guidelines recommend withholding antibiotics in selected patients, real-world prescribing patterns and associated outcomes remain unclear.

**Methods:**

We conducted a retrospective cohort study using nationwide Veterans Health Administration data (2016–2023) to identify emergency department visits for a first case of uncomplicated diverticulitis among patients discharged without admission. Low-risk diverticulitis was defined as age <65 years, Charlson Comorbidity Index <2, and white blood cell count <15 × 10^3^/µL. Outcomes included antibiotic prescribing trends, assessed using linear regression, and one-year hospitalization for diverticulitis. Analyses for one-year hospitalization were stratified by age and comorbidity burden to assess effect modification. Subdistribution hazard (Fine-Gray) models estimated adjusted hazard ratios, accounting for the competing risk of death.

**Results:**

Among 4,249 patients with low-risk diverticulitis (median age, 51 yr; 84.6% male), 4,068 (95.7%) received antibiotics. From 2016 to 2023, the proportion managed without antibiotics remained unchanged (approximately 4%), while β-lactam use increased and fluoroquinolone use declined. Antibiotic use was not significantly associated with a reduced risk of one-year hospitalization (adjusted hazard ratio, 0.99; 95% confidence interval, 0.54–1.84; *P* = .99). No significant differences were observed across age-comorbidity strata or antibiotic classes, although subgroup analyses may have been underpowered.

**Conclusions:**

Despite guideline recommendations, antibiotics were prescribed for most low-risk patients with uncomplicated diverticulitis, with no change over eight years. Clinical outcomes did not differ by antibiotic exposure, highlighting an opportunity to reduce unnecessary antibiotic use through outpatient stewardship interventions.

## Introduction

Outpatient settings account for 85–95% of all antibiotic prescriptions,^1^ and the frequency of these prescriptions has increased over time.^2^ However, approximately 30% of these prescriptions may be unnecessary or inappropriate.^3,4^ Such misuse contributes to antimicrobial resistance, adverse drug events, and increased healthcare costs.^5^ Although outpatient prescribing is a key target for antibiotic stewardship, most efforts have focused on respiratory, urinary, or skin and soft tissue infections.^6,7^ In contrast, less attention has been given to gastrointestinal infections, which represent an important opportunity for stewardship interventions.

Diverticulitis accounts for 450,000 diagnoses in the United States, with aggregate charges exceeding ten billion US dollars.^8,9^ Antibiotic treatment had long been the standard of care, but growing evidence suggests that non-antibiotic management is safe and reasonable for selected patients with uncomplicated diverticulitis.^10–16^ Recent clinical practice guidelines now recommend selective antibiotic use for low-risk patients, such as those with uncomplicated diverticulitis and without significant comorbidities.^9,17–21^

Despite these updates, the majority of patients discharged from the emergency department (ED) with diverticulitis still receive antibiotics.^22^ However, whether these prescribing patterns apply to low-risk populations remains unclear, and whether the effect of antibiotic treatment on recurrent diverticulitis outcomes differs across age-comorbidity risk subgroups is unknown. Therefore, we evaluated antibiotic prescribing patterns among low-risk patients with uncomplicated diverticulitis and assessed outcomes according to antibiotic exposure, using eight years of nationwide Veterans Health Administration (VHA) System data. Among the broader population of patients with uncomplicated diverticulitis, we further examined whether age-comorbidity risk subgroups modified the effect of antibiotic use on one-year hospitalization for recurrent diverticulitis. This expanded analysis aimed to inform clinicians about which patients across the broader spectrum of uncomplicated diverticulitis may be safely managed without antibiotics.

## Methods

### Study design and population

We conducted a retrospective cohort study (2016–2023) using the VHA Corporate Data Warehouse to identify ED visits for a first case of diverticulitis. Data were obtained through the Veterans Affairs Informatics and Computing Infrastructure.

We included patients who presented to the ED with an initial diagnosis of uncomplicated diverticulitis, defined by an International Classification of Diseases, 10th Revision (ICD-10) codes for diverticulitis without perforation or abscess (Supplementary Table 1), and were discharged directly from the ED without hospital admission. Patients were excluded if they were admitted to the hospital within 48 hours of the ED visit (on the assumption that admissions within this window were attributable to the index ED visit for diverticulitis), had a prior diagnosis of diverticulitis, had prior colorectal surgery, or had recent exposure (within 14 d prior to the ED visit) to antibiotics commonly used and considered appropriate for diverticulitis (β-lactams, fluoroquinolones, trimethoprim-sulfamethoxazole, and/or metronidazole).^23,24^

### Definitions

Age was categorized as younger (<65 yr) or elderly (≥65 yr), as older age is associated with higher diverticulitis-related mortality and atypical presentations,^24,25^ and the randomized trials supporting non-antibiotic management enrolled predominantly younger populations.^26^ Comorbidity burden was assessed using the original Charlson Comorbidity Index (CCI), with low comorbidity defined as CCI <2 and high comorbidity defined as CCI ≥2, to identify patients with potential immunocompromise, such as those with malignancy or moderate to severe chronic kidney or liver disease, for whom guidelines consistently recommend antibiotic treatment.^20,21,27^ White blood cell (WBC) count was categorized as <15 × 10^3^/μL or ≥15 × 10^3^/μL based on the American Gastroenterological Association.^20,21^ Body mass index (BMI) was categorized as underweight (18.5 kg/m^2^), normal weight (18.5–24.9 kg/m^2^), overweight (25.0–29.9 kg/m^2^), and obese (≥30.0 kg/m^2^).^28^

Low-risk diverticulitis was defined as patients meeting all of the following criteria: uncomplicated diverticulitis (as defined above), age <65 years, Charlson Comorbidity Index (CCI) <2, and WBC count <15 × 10^3^ /μL, based on recently updated clinical guidelines for diverticulitis management (Supplementary Table 2). We did not include C-reactive protein (CRP) in our definition, as it was not measured in 95.1% of the low-risk population in our cohort.

Patients were categorized as the antibiotic group if antibiotics commonly used and considered appropriate for diverticulitis (β-lactams, fluoroquinolones, trimethoprim-sulfamethoxazole, and/or metronidazole) were prescribed, and otherwise they were categorized as the no-antibiotic group. Within the antibiotic group, regimens were further categorized as follows: (a) β-lactam group, if amoxicillin/clavulanic acid (monotherapy or with metronidazole) or oral cephalosporins (cefuroxime, cefaclor, cephalexin, cefadroxil, cefdinir) plus metronidazole; (b) fluoroquinolone group, if ciprofloxacin or levofloxacin plus metronidazole, or moxifloxacin (monotherapy or with metronidazole); and (c) other antibiotics group, if all other combinations or regimens, including trimethoprim-sulfamethoxazole. Antibiotics dispensed on or within two days following the ED visit were attributed to the index encounter.

### Outcome measures

The outcomes included (i) trends in antibiotic prescriptions for patients with low-risk diverticulitis over the study period, (ii) hospitalization for recurrent diverticulitis within 365 days among patients with low-risk diverticulitis. Additionally, among all patients with uncomplicated diverticulitis, we examined whether age-comorbidity risk subgroups modified the effect of antibiotic use on one-year hospitalization for recurrent diverticulitis.

### Statistical analysis

Bivariable analyses were performed using Student’s *t*-test or Mann-Whitney *U*-test for continuous variables, and *χ* ^2^ tests or Fisher’s exact tests for categorical variables.

For prescribing trends among patients with low-risk diverticulitis, we modeled the proportion of each antibiotic group among all prescriptions using linear regression, with calendar quarter (Q1 2016–Q4 2023) as the independent variable. Analyses were conducted for the proportion of patients receiving each antibiotic class (β-lactam group, fluoroquinolone group, or other antibiotics group) and the no-antibiotic group and the mean duration of a single course of antibiotic therapy. Regression coefficients representing slope estimates were reported to describe changes in prescribing patterns over time. The linearity assumption was evaluated by visual inspection of residuals plotted against fitted values and by sequential ANOVA *F*-tests comparing linear and quadratic polynomial models.

For the risk of one-year hospitalization among those with low-risk diverticulitis, we compared outcomes between the antibiotic and no-antibiotic groups. Missing values in baseline covariates were addressed using multiple imputation with chained equations, generating twenty imputed datasets. We fit subdistribution hazard (Fine-Gray) models to estimate hazard ratios (HRs) for one-year hospitalization due to recurrent diverticulitis, accounting for the competing risk of death. For the multivariable analysis, WBC count was categorized as normal (4.0–11.9 × 10^3^/μL) or abnormal (<4.0 or ≥12.0 × 10^3^/μL) according to the Sepsis-3 consensus definitions,^29^ as we hypothesized that providers’ antibiotic prescribing behavior might depend on WBC count. Patient demographics, BMI, CCI, and WBC count were adjusted. Results were pooled using Rubin’s rules. As sensitivity analyses, we performed a complete-case analysis and repeated the primary analysis restricted to patients who underwent computed tomography (CT) imaging within 48 hours of the index ED visit.

Among patients with uncomplicated diverticulitis, as defined by ICD-10 codes, we examined whether age-comorbidity risk subgroups modified the effect of antibiotic use on one-year hospitalization for recurrent diverticulitis. The following groups were created based on age and comorbidity: (a) young/low comorbidity (age <65 yr and CCI <2), (b) elderly/low comorbidity (age ≥65 yr and CCI <2), (c) young/high comorbidity (age <65 yr and CCI ≥2), and (d) elderly/high comorbidity (age ≥65 yr and CCI ≥2). We tested for effect modification by including interaction terms between antibiotic use and age-comorbidity risk groups in multivariable Fine-Gray models. The overall significance of these multiplicative interaction terms was evaluated using Wald tests with robust variance estimates, pooled across imputed datasets.

In subgroup analyses, we compared individual antibiotic classes (β-lactam and fluoroquinolone groups) with the no-antibiotic group using the same analytic strategy.

All analyses were performed two-tailed, with alpha <0.05 considered statistically significant. Statistical analyses were conducted using R version 4.5.2 (R Foundation for Statistical Computing, Vienna, Austria).

## Results

**Figure 1.**
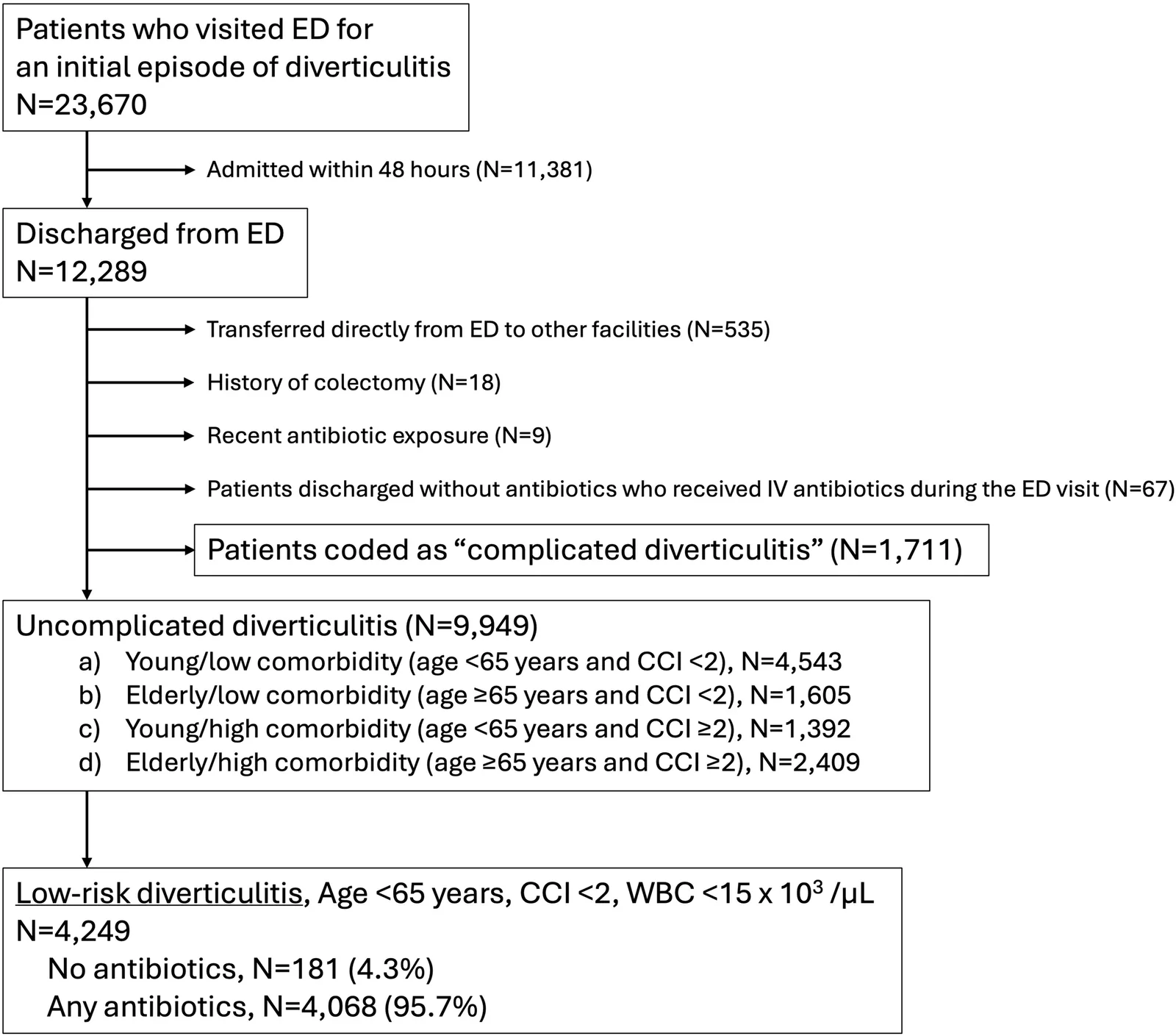
Flow diagram of eligibility for inclusion in the study. Abbreviations: CCI, Charlson Comorbidity Index; CRP, C-reactive protein; ED, emergency department; WBC, white blood cell.

Among 23,670 patients who visited the ED for an initial case of diverticulitis, 12,289 were discharged from the ED. Of these, 9,886 were classified as having uncomplicated diverticulitis based on the ICD-10 code (Figure 1). Among them, 4,249 (median age, 51 yr; 84.6% male) met criteria for low-risk diverticulitis (Figure 1). Only 181 (4.3%) were treated without antibiotics. Among the 4,068 patients (95.7%) who received antibiotics, 1,594 (39.2%) were classified into the β-lactam group, 2,114 (52.0%) into the fluoroquinolone group, and 360 (8.8%) into the other antibiotics group. Within the β-lactam, fluoroquinolone, and other antibiotics groups, amoxicillin/clavulanate monotherapy, ciprofloxacin or levofloxacin plus metronidazole, and trimethoprim-sulfamethoxazole plus metronidazole accounted for 1,396 (87.6%), 2,093 (99.0%), and 140 (38.9%) patients, respectively.

Baseline characteristics of patients with low-risk diverticulitis are summarized in Table 1. As expected based on our low-risk definition (age <65 yr, CCI <2, WBC count <15 × 10^3^ /μL), the cohort excluded patients with most major comorbidities, and the maximum age was 64 years. The mean WBC count was significantly lower in the no-antibiotic group than in the antibiotic group.

**Table 1.** Baseline characteristics of patients with low-risk diverticulitis discharged from the emergency department without admission, by whether antibiotics were prescribed Table 1 long description.

	No antibiotics (N = 181)	Any antibiotics (N = 4,068)	*P*-value
Age, y	50 [40–57]	51 [42–58]	.10
Male gender	148 (81.8)	3,444 (84.7)	.34
BMI, kg/m^2^ (N = 3,841)	31.7 [27.9–35.3]	32.4 [29.1–36.4]	.09
Race (N = 3,805)			.033
White	109 (71.7)	2,803 (76.7)	
Black or African American	35 (23.0)	676 (18.5)	
Asian	4 (2.6)	25 (0.7)	
Multi-race	1 (0.7)	66 (1.8)	
Other	3 (2.0)	83 (2.3)	
Co-morbidities/past medical history			
Myocardial infarction	1 (0.6)	17 (0.4)	1.00
Congestive heart failure	2 (1.1)	25 (0.6)	.74
Peripheral vascular disease	2 (1.1)	48 (1.2)	1.00
Cerebrovascular accident or transient ischemic attacks	5 (2.8)	41 (1.0)	.06
Dementia	0 (0.0)	2 (0.0)	1.00
Chronic pulmonary disease	24 (13.3)	558 (13.7)	.95
Connective tissue disease	3 (1.7)	31 (0.8)	.37
Peptic ulcer disease	0 (0.0)	28 (0.7)	.52
Chronic hepatitis or cirrhosis (mild)	7 (3.9)	228 (5.6)	.40
Chronic hepatitis or cirrhosis (moderate to severe)	0 (0.0)	0 (0.0)	NA
Diabetes (uncomplicated)	11 (6.1)	307 (7.5)	.56
Diabetes (complicated)	0 (0.0)	0 (0.0)	NA
Chronic kidney diseases	0 (0.0)	0 (0.0)	NA
Malignancy	0 (0.0)	0 (0.0)	NA
HIV/AIDS	0 (0.0)	0 (0.0)	NA
Charlson Comorbidity Index=1 (reference: 0)	55 (30.4)	1,285 (31.6)	.80
White blood cells count, x 10^3^ /μL (N = 3,936)	8.1 [6.4–10.2]	9.6 [7.7–11.6]	<.001
C-reactive protein, mg/dL (N = 207)	0.4 [0.2–4.5]	3.3 [1.1–6.8]	.031

Data are presented as number (%) or median [interquartile range]. Abbreviations: AIDS, acquired immunodeficiency syndrome; BMI, body mass index; HIV, human immunodeficiency virus.

### Outcomes: prescribing trends

**Figure 2.**
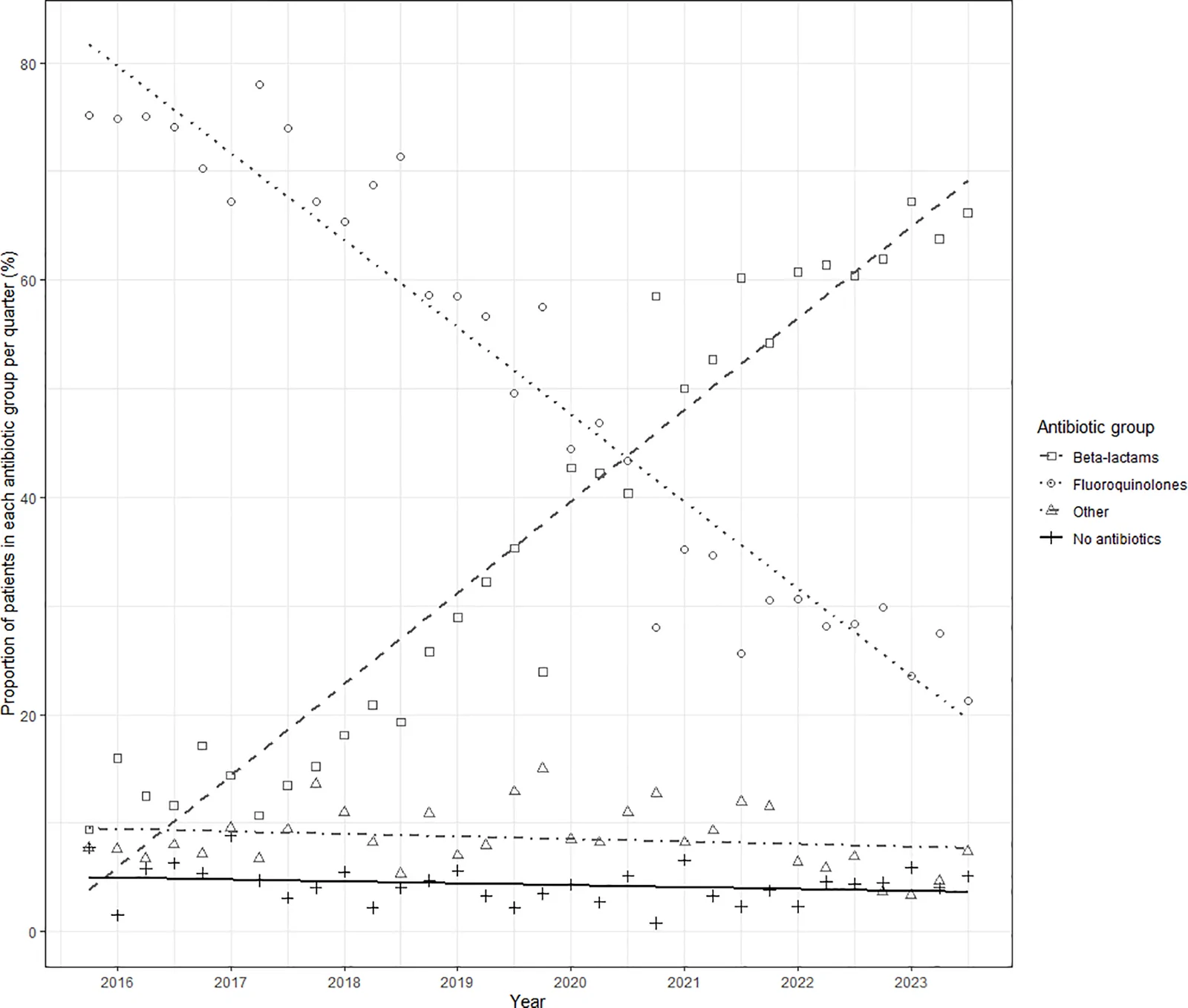
Trends in the proportion of patients prescribed each antibiotic group for low-risk diverticulitis per quarter.

From Q1 2016 to Q4 2023, the number of patients presenting with low-risk diverticulitis increased slightly and lineary over time (slope, 0.79 [95% CI: +0.20 to +1.38] patient per quarter; *p* = .011). The proportion of patients not prescribed antibiotics remained stable throughout the study period (−0.04% [95% CI: −0.11% to +0.02%] per quarter, *p* = .20) (Figure 2). Prescribing patterns revealed shift in β-lactam use increased steadily (slope, +2.11% [95% CI +1.90% to +2.32%]; *p* < .001), while fluoroquinolone use declined consistently (slope, −2.01% [95% CI −2.22% to −1.79%]; *p* < .001); residual diagnostics confirmed linearity for both trends. Among the 97.9% of patients for whom antibiotic duration was available, the median duration was 10 days (interquartile range, 7–10 d) across the study period, and the duration of antibiotic prescriptions declined modestly and linearly over time (slope,−0.028 [95% CI: −0.037 to −0.019] days per quarter; *p* < .001), corresponding to an approximately 0.9-day reduction per antibiotic course over eight years (Supplementary Figure 1).

### Outcomes: the risk of one-year hospitalization

Missing data included race (444, 10.4%), BMI (408, 9.6%), and WBC count (313, 7.5%).

Within 365 days, 11 patients (6.1%) in the no-antibiotic group and 245 patients (6.0%) in the antibiotic group were hospitalized for diverticulitis. Antibiotic prescription was not significantly associated with a reduced hazard of one-year admission due to diverticulitis (univariable model: HR .99, 95% CI 0.54–1.81, *p* = .97; multivariable model: HR 0.99, 95% CI 0.54–1.84, *p* = .99; Table 2). A complete-case sensitivity analysis yielded consistent results (univariable model: HR 0.99, 95% CI 0.54–1.81, *p* = .98; multivariable model: HR 1.07, 95% CI 0.47–2.44, *p* = .86). Among patients with low-risk diverticulitis, 3,677 of 4,249 (86.5%) underwent CT imaging. In the sensitivity analysis restricted to these CT-performed cases, results were consistent with the primary analysis (univariable model: HR 1.13, 95% CI 0.50–2.54, *p* = .77; multivariable model: HR 1.13, 95% CI 0.50–2.60, *p* = .76). In the multivariable model, Black or African American race was associated with a higher hazard of one-year admission due to diverticulitis compared with White race (adjusted HR 1.42, 95% CI 1.05–1.92, *p* = .023).

**Table 2.** The risk of one-year hospital admission for diverticulitis among low-risk cases comparing no-antibiotic and any antibiotic groups Table 2 long description.

	One-year hospital admission for diverticulitis	
Unadjusted analysis	Hazard ratios (95% CIs)	*p*-value
No-antibiotics (*N*=181)	Reference	N/A
Any antibiotics (*N*=4,068)	0.99 (0.54–1.81)	.97
Adjusted analysis	Hazard ratios (95% CIs)	*p*-value
No-antibiotics (*N*=181)	Reference	N/A
Any antibiotics (*N*=4,068)	0.99 (0.54–1.84)	.99
Age (years)	0.99 (0.98–1.00)	.06
Gender, Male	1.00 (0.71–1.41)	.98
Race		
White	Reference	N/A
Black or African American	1.42 (1.05–1.92)	.023
Other race	0.52 (0.22–1.24)	.14
BMI, kg/m^2^		
Normal (18.5–24.9)	Reference	N/A
Underweight (<18.5)	4.57 (0.50–41.70)	.18
Overweight (25.0–29.9)	2.12 (0.91–4.91)	.083
Obese (≥30.0)	2.15 (0.95–4.89)	.067
CCI=1 (reference: 0)	1.24 (0.96–1.61)	.10
WBC count		
Normal (4.0–11.9)	Reference	N/A
Abnormal (<4.0 or ≥12.0)	1.13 (0.83–1.52)	.44

Subdistribution hazard ratios from Fine-Gray models accounting for death. Multiple imputation was used for missing values in race, BMI, and WBC count; see Methods. Abbreviations: BMI, body mass index; CCI, Charlson Comorbidity Index; CI, confidence interval; WBC, white blood cell.

When hospitalization risk among all uncomplicated patients was stratified by age-comorbidity burden (young/low comorbidity, N = 4,543; elderly/low comorbidity, N = 1,605; young/high comorbidity, N = 1,605; and elderly/high comorbidity, N = 2,409) to estimate effect modification by risk groups, no subgroup demonstrated a significantly decreased hazard of one-year admission due to diverticulitis associated with prescribing antibiotics (Table 3). Although point estimates suggested some numerical variation across subgroups (HRs ranging from 0.57 to 1.07), the overall test for interaction was not statistically significant (Wald test, *p* = .67), indicating no strong evidence that antibiotic effects differed by patient age and comorbidity status.

**Table 3.** Effect of antibiotic use on one-year hospitalization for recurrent diverticulitis by age-comorbidity subgroups among patients with uncomplicated diverticulitis Table 3 long description.

Subgroup	No antibiotics, N	Any antibiotics, N	Total patients, N	Events, N	Adjusted HR (95% CIs)*	*p*-value
Young patients with low comorbidity	187	4,356	4,543	281	1.07 (0.58–1.97)	.83
Elderly patients with low comorbidity	73	1,532	1,605	78	0.69 (0.28–1.73)	.43
Young patients with high comorbidity	72	1,320	1,392	84	0.57 (0.25–1.30)	.18
Elderly patients with high comorbidity	130	2,279	2,409	134	0.95 (0.46–1.94)	.89

* Reference group: no antibiotics. Adjusted for Age, Gender, Race, BMI, CCI, and WBC count. *P* value for multiplicative interaction (Wald test) =0.67. Abbreviations: BMI, body mass index; CCI, Charlson Comorbidity Index; CI, confidence interval; HR, hazard ratio; WBC, white blood cell.

### Subgroup analyses

In subgroup analyses stratified by antibiotic class (β-lactam and fluoroquinolone groups), neither β-lactams nor fluoroquinolones were associated with a significantly altered risk of one-year hospitalization compared with the no-antibiotic group in subdistribution hazard (Fine-Gray) models (Supplementary Table 3).

## Discussion

In this U.S. nationwide retrospective cohort study, we found that antibiotics were prescribed for more than 95% of low-risk patients with uncomplicated diverticulitis, despite changes in guideline recommendations, and that the proportion of patients managed without antibiotics remained stable over eight years. Among patients who received antibiotics, prescribing patterns shifted significantly, with increased use of β-lactams and decreased use of fluoroquinolones, with a modest reduction in duration of therapy. Adjusted hazard ratios for one-year hospital admission due to diverticulitis did not differ significantly between the no-antibiotic and antibiotic groups, consistent with prior randomized trials. When hospitalization risk in all uncomplicated patients was stratified by age-comorbidity burden, no obvious effect modification was observed, and no subcategory demonstrated a statistically significant association between antibiotic use and lower hazard of one-year hospitalization, supporting the potential safety of selective non-antibiotic management across diverse patient profiles.

Our findings highlight a persistent gap between updated guideline recommendations on selective antibiotic use for low-risk diverticulitis and real-world prescribing patterns. A prior survey of ED providers, general practitioners, and surgeons identified two main reasons for avoiding non-antibiotic management of uncomplicated diverticulitis: (i) concerns about treatment failure (70%) and (ii) lack of awareness of the recommendations (67%).^30^ Another survey of surgeons showed that only 26% had incorporated selective antibiotic use for low-risk patients into their practice, and 50% disagreed that the updated guidelines were likely to change their practice. [9] These challenges may be compounded by vague or inconsistent definitions of “low-risk” across guidelines. Some guidelines rely solely on CT findings, while others incorporate symptoms or laboratory data (Supplementary Table 2). In addition, recent recommendations for selective imaging to guide the diagnosis of diverticulitis further complicate guideline implementation,^9^ given provider disinclination to prescribe antibiotics without a CT confirmation of uncomplicated diverticulitis. The stable rate of no-antibiotic prescribing in our U.S. multicenter cohort suggests that barriers to adopting no-antibiotic management have persisted in routine clinical practice. Further studies are needed to clarify safe thresholds for no-antibiotic management and to promote guideline-concordant practices among frontline providers.

Our cohort confirmed increased use of β-lactams and decreased use of fluoroquinolones. The reduction in fluoroquinolone use likely reflects the 2016 United States Food and Drug Administration (FDA) safety warnings, which recommended restricting fluoroquinolones due to the risk of serious adverse effects.^31,32^ Several studies have demonstrated that fluoroquinolones are not superior to β-lactams for management of diverticulitis,^20,26,33^ consistent with our findings. While prescribing behavior appears to have shifted from fluoroquinolones to β-lactams and overall treatment durations have modestly shortened, the rate of no-antibiotic management remained low throughout the study period. Given that no-antibiotic management was not associated with significantly increased hospitalization for recurrent diverticulitis in our low-risk cohort, prospective stewardship interventions are warranted to safely reduce unnecessary antibiotic prescriptions.

Our results may suggest that selective no-antibiotic management may be safe even for older patients or those with higher comorbidity burdens. Analyses stratified by age and comorbidity demonstrated no clear association between antibiotic prescription and the risk of one-year admission, supporting the potential safety of selective no-antibiotic management across diverse patient profiles. However, the precise thresholds for safely withholding antibiotics remain unclear. In fact, point estimates of adjusted hazard ratios among patients treated with antibiotics were lower in the young/high comorbidity and elderly/low comorbidity groups relative to the elderly/high comorbidity group. This pattern suggests that certain older or more comorbid populations could potentially benefit from antibiotic treatment, although the boundary between those who may benefit and those who may not remains unclear. These findings may also reflect limitations of age or CCI as risk indicators for recurrent diverticulitis or potential misclassification from ICD coding. Further studies are needed to better define safe boundaries for no-antibiotic management across varying risk profiles.

Black or African American patients demonstrated an increased hazard of one-year hospital admission for diverticulitis compared to White patients. This finding aligns with exisiting literature documenting racial disparities in diverticulitis outcomes. A recent systematic review found that the majority of studies reported worse outcomes in Black compared to White patients, including higher readmission and mortality rates.^34^ However, insurance status and socioeconomic factors appear to be stronger predictors than race alone, suggesting these disparities reflect systemic barriers to care rather than biological differences.^34^

This study has several limitations. Residual confounding from unmeasured variables cannot be excluded; providers likely prescribed antibiotics to sicker patients based on factors not captured in administrative data, which would most plausibly bias the estimate toward the null. Additionally, the small no-antibiotic group yielded wide confidence intervals, though the point estimate is consistent with prior randomized trials.^10–12,15^ Our cohort was predominantly male, potentially limiting generalizability, although prior studies have not demonstrated sex-specific differences in outcomes.^35^ We relied on ICD codes without access to CT imaging data, though a sensitivity analysis restricted to CT-performed cases revealed results. Incorporating CT findings via natural language processing would further improve diagnostic accuracy, as CT remains the diagnostic gold standard.^20,21^ When stratified by age-comorbidity burden, the non-significant hazard ratios may be attributed to small sample sizes within each group. The number of events per variable in our multivariable model (n = 37) was adequate for covariates with moderate prevalence but may have limited precision in estimating effects of covariates with sparse categories.^36^ Adherence to prescribed antibiotics after the ED visit was also unknown.

In conclusion, antibiotics were prescribed for most low-risk patients with uncomplicated diverticulitis in the VHA, with increasing use of β-lactams and decreasing use of fluoroquinolones over time. Many antibiotic prescriptions in this population may be unnecessary, as clinical outcomes did not differ significantly between patients treated with or without antibiotics, consistent with prior randomized trials, though the small no-antibiotic group limits the precision of this comparison in our cohort. These findings may also be relevant to older patients and those with higher comorbidity burdens, but further studies are needed to refine definitions of low-risk diverticulitis and to develop strategies that safely reduce unnecessary antibiotic use.

## Supporting information

10.1017/ash.2026.10884.sm001 Hasegawa et al. supplementary material 1 Hasegawa et al. supplementary material

10.1017/ash.2026.10884.sm002 Hasegawa et al. supplementary material 2 Hasegawa et al. supplementary material

## Supplementary material

The supplementary material for this article can be found at https://doi.org/10.1017/ash.2026.10884.

## Table 1. Long description

A table comparing baseline characteristics of patients with low-risk diverticulitis by antibiotic prescription status. The table has 18 rows and 5 columns. Column headers are Age, Male gender, BMI, Race, Co-morbidities/past medical history. Row labels include Age, Male gender, BMI, Race, and various co-morbidities. The table compares patients who did not receive antibiotics (N=181) with those who received any antibiotics (N=4,068). Notable columns include Age, Male gender, BMI, and Race. The table also lists various co-morbidities and their prevalence in both groups. The P-value column indicates the statistical significance of the differences between the groups.

Navigate back to Table 1..

## Table 2. Long description

A table comparing the risk of one-year hospital admission for diverticulitis among low-risk cases between no-antibiotic and any antibiotic groups. The table has 18 rows and 4 columns. The columns are labeled ‘Unadjusted analysis’, ‘Hazard ratios (95% CIs)’, ‘p-value’, ‘Adjusted analysis’, ‘Hazard ratios (95% CIs)’, and ‘p-value’. The rows are labeled with different variables such as ‘No-antibiotics (N=181)’, ‘Any antibiotics (N=4,068)’, ‘Age (years)’, ‘Gender, Male’, ‘Race’, ‘White’, ‘Black or African American’, ‘Other race’, ‘BMI, kg/m^2’, ‘Normal (18.5–24.9)’, ‘Underweight (<18.5)’, ‘Overweight (25.0–29.9)’, ‘Obese (≥30.0)’, ‘CCI=1 (reference: 0)’, ‘WBC count’, ‘Normal (4.0–11.9)’, and ‘Abnormal (<4.0 or ≥12.0)’. Each row provides hazard ratios, 95% confidence intervals, and p-values for the respective variables.

Navigate back to Table 2..

## Table 3. Long description

A table comparing the effect of antibiotic use on one-year hospitalization for recurrent diverticulitis by age-comorbidity subgroups. The table has five columns: Subgroup, No antibiotics (N), Any antibiotics (N), Total patients (N), Events (N), Adjusted HR (95% CIs), and p-value. It includes four subgroups: Young patients with low comorbidity, Elderly patients with low comorbidity, Young patients with high comorbidity, and Elderly patients with high comorbidity. Each row provides the number of patients without antibiotics, with any antibiotics, total patients, events, adjusted hazard ratio with confidence intervals, and p-value for each subgroup. The table shows that no subgroup demonstrated a significantly decreased hazard of one-year admission due to diverticulitis associated with prescribing antibiotics.

Navigate back to Table 3..
