# Supplementary material for: Antibiotic prescribing trends and outcomes in outpatient low-risk diverticulitis: a retrospective analysis in Veterans Health Administration between 2016 and 2023
Source: Antimicrob Steward Healthc Epidemiol. 2026 Oct 1;6(1):e309. doi: 10.1017/ash.2026.10884 (PMC13760418; doi:10.1017/ash.2026.10884)
Supplement: Supplementary file 1 [file S2732494X26108845sup001.docx]

Supplementary figure 1. Trends in mean duration of antibiotic therapy for low-risk diverticulitis per quarter.
