# Supplementary material for: Antibiotic prescribing trends and outcomes in outpatient low-risk diverticulitis: a retrospective analysis in Veterans Health Administration between 2016 and 2023
Source: Antimicrob Steward Healthc Epidemiol. 2026 Oct 1;6(1):e309. doi: 10.1017/ash.2026.10884 (PMC13760418; doi:10.1017/ash.2026.10884)
Supplement: Supplementary file 2 [file S2732494X26108845sup002.docx]

**Supplementary table 1.** ICD codes used to identify patients with diverticulitis-related diagnostic codes, with percutaneous drain or colectomy (for any indication)

a. Diverticulitis ICD-9 code:

i) Uncomplicated: 562.11, 562.13

ii) Complicated: 562.11, 562.13

b. Diverticulitis ICD-10 code

i) Uncomplicated: K57.32, K57.33, K57.52, K57.53, K57.9, K57.93

ii) Complicated: K57.20, K57.21, K57.40, K57.41, K57.80, K57.81

c. Colectomy ICD-9-CM procedure

1733, 1734, 1735, 1736, 1739, 4573, 4574, 4575, 4576, 4579, 4581, 4582, 4583

d. Colectomy ICD-10-PCS procedure:

0DBE0ZZ, 0DBE3ZZ, 0DBE4ZZ, 0DBE7ZZ, 0DBE8ZZ,

0DBF0ZZ, 0DBF3ZZ, 0DBF04ZZ, 0DBF7ZZ, 0DBF8ZZ,

0DBG0ZZ, 0DBG3ZZ, 0DBG4ZZ, 0DBG7ZZ, 0DBG8ZZ, 0DBGFZZ,

0DBH0ZZ, 0DBH3ZZ, 0DBH4ZZ, 0DBH7ZZ, 0DBH8ZZ,

0DBK0ZZ, 0DBK3ZZ, 0DBK4ZZ, 0DBK7ZZ, 0DBK8ZZ,

0DBL0ZZ, 0DBL3ZZ, 0DBL4ZZ, 0DBL7ZZ, 0DBL8ZZ, 0DBLFZZ,

0DBM0ZZ, 0DBM3ZZ, 0DBM4ZZ, 0DBM7ZZ, 0DBM8ZZ, 0DBMFZZ,

0DBN0ZZ, 0DBN3ZZ, 0DBN4ZZ, 0DBN7ZZ, 0DBN8ZZ, 0DBNFZZ,

0DTE0ZZ, 0DTE4ZZ, 0DTE7ZZ, 0DTE8ZZ,

0DTF0ZZ, 0DTF4ZG, 0DTF4ZZ, 0DTF7ZZ, 0DTF8ZZ,

0DTG0ZZ, 0DTG4ZZ, 0DTG7ZZ, 0DTG8ZZ, 0DTGFZZ,

0DTK0ZZ, 0DTK4ZZ, 0DTK7ZZ, 0DTK8ZZ,

0DTL0ZZ, 0DTL4ZG, 0DTL4ZZ, 0DTL7ZZ, 0DTL8ZZ, 0DTLFZZ,

0DTM0ZZ, 0DTM4ZZ, 0DTM7ZZ, 0DTM8ZZ, 0DTMFZZ,

0DTN0ZZ, 0DTN4ZZ, 0DTN7ZZ, 0DTN8ZZ, 0DTNFZZ

**Supplementary table 2.** Definitions of low-risk diverticulitis in different guidelines.

| Association | Year | Definition of low-risk patients |
| --- | --- | --- |
| The index study | 2025 | Excludes perforation/abscess by ICD codes, CRP ≥14 mg/dL, WBC ≥15 x 10^3^ /μL, CCI ≥2 |
| American College of Physician | 2022 | Left-sided. Excludes severe symptoms (>5 days, vomiting), systemic comorbidity, high CRP (>140 mg/L), CT findings (air, fluid, etc) |
| American Gastroenterological Association | 2021 | Excludes abscess, peritonitis, obstruction, stricture, or fistula on CT, or comorbidities, frailty, refractory symptoms, vomiting, CRP >140 mg/L, or WBC >15 x 10^3^ /μL |
| American Society of Colon and Rectal Surgeons | 2020 | Left-sided. Excludes uncontained free perforation with a systemic inflammatory response, fistula, abscess, stricture, or obstruction on CT |
| World Society of Emergency Surgery  European Society of Coloproctology  European Association of Endoscopic Surgery and Society of American Gastrointestinal and Endoscopic | 2020  2020  2019 | Left-sided. Excludes pericolic air bubbles, abscess, or distant gas on CT  Left-sided. Hinchey classification Ia (Excludes Hinchey $\geq$Ib: abscess, free perforations, or peritonitis)  Left-sided. Hinchey classification Ia (Excludes Hinchey $\geq$Ib: abscess, free perforations, or peritonitis) |

Abbreviations: CCI, Charlson Comorbidity Index; CRP, C-reactive protein; CT, computed tomography; ICD, International Classification of Diseases; WBC, white blood cell.

**Supplementary table 3.** Subgroup analyses: one-year hospitalization risk for diverticulitis among low-risk diverticulitis cases comparing no-antibiotic and each antibiotic group using subditribution hazard (Fine-Gray) models.

|  | One-year hospital admission for diverticulitis | |
| --- | --- | --- |
| Unadjusted Analysis | **Hazard Ratios (95% CIs)** | **p-value** |
| No-antibiotics (N=181) | Reference | N/A |
| β-lactams (N=1,594)  Fluoroquinolones (N=2,114)  Other antibiotics (N=360) | 0.99 (0.53-1.85)  1.00 (0.54-1.86)  0.91 (0.44-1.91) | 0.98  0.99  0.81 |
|  |  |  |
| Adjusted Analysis | **Hazard Ratios (95% CIs)** | **p-value** |
| No-antibiotics (N=181) | Reference | N/A |
| β-lactams (N=1,594)  Fluoroquinolones (N=2,114)  Other antibiotics (N=360) | 1.01 (0.54-1.90)  1.01 (0.54-1.89)  0.91 (0.43-1.93) | 0.97  0.98  0.81 |
| Age  Gender, Male  Race  White  Black or African American  Other  BMI, kg/m^2^  Normal (18.5-24.9)  Underweight (<18.5)  Overweight (25.0-29.9)  Obese (≥30.0)  CCI = 1  WBC count  Normal (4.0-11.9)  Abnormal (<4.0 or ≥12.0) | 0.99 (0.98-1.00)  1.00 (0.71-1.41)  Reference  1.39 (1.02-1.90)  0.53 (0.22-1.24)  Reference  4.79 (0.54-42.34)  2.10 (0.91-4.87)  2.15 (0.95-4.88)  1.24 (0.96-1.61)  Reference  1.12 (0.82-1.53) | 0.06  1.00  N/A  0.037  0.14  N/A  0.16  0.08  0.07  0.10  N/A  0.47 |
| Abbreviations: BMI, body mass index; CCI, Charlson Comorbidity Index; CI, confidence interval; WBC, white blood cell. | | |
|  |  |  |
